# Categorization of Novel Research Ideas Regarding Adolescent Idiopathic Scoliosis Generated by Artificial Intelligence

**DOI:** 10.7759/cureus.74574

**Published:** 2024-11-27

**Authors:** Christian J Leonardo, Kevin Melcer, Steven H Liu, David E Komatsu, James M Barsi

**Affiliations:** 1 Department of Orthopedic Surgery, Stony Brook University, Stony Brook, USA

**Keywords:** adolescent idiopathic scoliosis (ais), aritifical intelligence, chatgpt-3.5, ortho-surgery, research design

## Abstract

Background

The generation of innovative research ideas is crucial to advancing the field of medicine. As physicians face increasingly demanding clinical schedules, it is important to identify tools that may expedite the research process. Artificial intelligence may offer a promising solution by enabling the efficient generation of novel research ideas. This study aimed to assess the feasibility of using artificial intelligence to build upon existing knowledge by generating innovative research questions.

Methods

A comparative evaluation study was conducted to assess the ability of AI models to generate novel research questions. The prompt "research ideas for adolescent idiopathic scoliosis" was input into ChatGPT 3.5, Gemini 1.5, Copilot, and Llama 3. This resulted in an output of several research questions ranging from 10 questions to 14 questions. A keyword-friendly modified version of the AI-generated responses was searched in the PubMed database. Results were limited to manuscripts published in the English language from the year 2000 to the present. Each response was then cross-referenced to the PubMed search results and assigned an originality score of 0-5, with 0 being the most original and 5 being not original at all, by adding one numerical value for each paper already published on the topic. The mean originality scores were calculated manually by summing the originality scores from all the responses from each AI model and then dividing that sum by the respective number of prompts generated by the AI. The standard deviation of the originality scores for each AI was calculated using the standard deviation function (STDEV) function in Google Sheets (Google, Mountain View, California). Each AI was also evaluated on its percent novelty, the percentage of total generated responses that yielded an originality score of 0 when searched in PubMed.

Results

Each AI produced varying numbers of research prompts that were inputted into PubMed. The mean originality scores for ChatGPT, Gemini, Copilot, and Llama were 4.2 ± 1.9, 4.1 ± 1.3, 4.0 ± 1.6, and 3.8 ± 1.7, respectively. Of ChatGPT's 12 prompts, 16.67% were completely novel (no prior research had been conducted on the topic provided by the AI model). 10.00% of Copilot's 10 prompts were completely novel, and 8.33% of Llama's 12 prompts were completely novel. None of Gemini's fourteen responses yielded an originality score of 0.

Conclusions

Our findings demonstrate that ChatGPT, Llama, and Copilot are capable of generating novel ideas in orthopaedics research. As these models continue to evolve and become even more refined with time, physicians and scientists should consider incorporating them when brainstorming and planning their research studies.

## Introduction

The capacity of physicians to generate innovative research ideas is crucial for enhancing patient outcomes and propelling the field of orthopedic surgery forward. Previous research has repeatedly shown that rigorous research is not only academically necessary but vital to improving patient care [1-2]. However, as the scope of orthopedics expands and physicians face increasingly demanding clinical schedules, less time is available for academic endeavors. Artificial intelligence tools like ChatGPT, Gemini, Copilot, and Llama may offer a promising solution by efficiently generating novel research ideas. 

In the previous two decades, there has been a remarkable 11-fold surge in internet usage [3]. With this heightened reliance on the internet, companies have turned their focus toward not only enhancing existing search engine capabilities but in creating new ones, such as artificial intelligence. In response to user-generated input (e.g., questions or prompts), artificial intelligence (AI) models such as ChatGPT generate human-like responses to the input with minimal human intervention. While ChatGPT has been among the most prominent publicly available AI models [4], other AI models have also risen in usage and popularity, including Gemini, Copilot, and Llama. With the development of all these models, the potential for their use in healthcare has been made increasingly apparent by their capacities for early disease detection, medical decision-making, and facilitating clinical workflow [5-7]. 

Despite the relatively recent innovation of these AI models, they have garnered significant attention and use in research, as evidenced by over 1000 citations identified by the keyword "ChatGPT" on PubMed as of August 2023 [8]. Alongside ChatGPT, other AI platforms such as Gemini and Copilot have also been utilized, with a recent study showcasing their ability to generate literature reviews in research in dermatology and musculoskeletal radiology [9-10]. In addition, previous studies have emphasized the utility of these models in patient communication and education, writing research papers and literature reviews [3, 8-12], and even the expansion of AI into the clinical setting by helping to diagnose diseases and assist with genetic engineering [13-15]. 

The role of AI in the clinical aspect of medicine is highlighted by a recent study that found AI to be capable of predicting extended postoperative opioid use following total knee arthroplasty [16]. While the utility of AI is certainly far-reaching in both clinical and academic medicine, there has yet to be an investigation into the use of artificial intelligence for generating novel research ideas in orthopedics and orthopedic surgery. Our study aims to assess the feasibility of leveraging the capabilities of artificial intelligence models to build upon existing knowledge to generate innovative research ideas in orthopedics. 

## Materials and methods

A comparative evaluation study was conducted to assess the ability of AI models to generate novel research questions. We inserted the prompt "research ideas for adolescent idiopathic scoliosis" into ChatGPT3.5 (Open AI), Gemini 1.5 (Google AI), Copilot (Microsoft AI), and Llama 3 (Meta AI). Each model responded with a definition of adolescent idiopathic scoliosis and a varying number of research ideas, ranging from 10 (Copilot) to 14 (Gemini). Responses were then modified to optimize keyword search results on PubMed because the generated responses were too long to return search results. For example, ChatGPT's first research idea was "Genetic and Molecular Factors: Investigate the genetic and molecular mechanisms underlying adolescent idiopathic scoliosis (AIS). Explore the role of specific genes, genetic markers, or epigenetic factors in the development and progression of scoliosis. This could involve genome-wide association studies (GWAS) or molecular biology techniques". Accordingly, we inserted the following modified prompt into PubMed: "Genetic and molecular factors of adolescent idiopathic scoliosis". After inputting these keywords into PubMed, we counted how many papers had been published on the topic in the English language since the year 2000. Next, we assigned each topic an originality score between zero and five, with zero being the most original and five being not original at all. We determined the originality score by counting how many studies had already been published on the topic. For every paper that had already been published, the score increased by one numerical value until the numerical value of five was reached. The mean originality score for each AI was calculated manually by calculating the sum of the originality scores across the prompts generated by each AI and then dividing that sum by the number of prompts that were generated. The standard deviation was calculated using the "STDEV" function on Google Sheets (Google, Mountain View, California). The percent novelty was also calculated by dividing the number of completely novel prompts (with an originality score of 0) by the total number of prompts given and multiplying that proportion by 100%. 

This study did not involve human subjects and was therefore exempt from our institution's IRB. 

## Results

The prompt "research ideas for adolescent idiopathic scoliosis" elicited varying numbers of responses from each artificial intelligence model (Tables 1-4). An originality score of 5 was assigned to topics where there were at least five matching results on the PubMed database, indicating a low level of novelty. Conversely, an originality score of 0 was assigned to topics with absolutely no matches on PubMed, indicating complete novelty. Each AI model's mean originality scores were tabulated (Table 5). For ChatGPT, Gemini, Copilot, and Llama, the mean +/- standard deviation originality scores were 4.2 ± 1.9, 4.1 ± 1.3, 4.0 ± 1.6, and 3.8 ± 1.7, respectively. Across these four mean originality scores, the average mean originality score was 4.0. 

**Table 1:** Results from the ChatGPT query AIS - adolescent idiopathic scoliosis

ChatGPT responses	PubMed input	Originality score
Patient-Specific Treatment Plans: Develop algorithms or predictive models that can help tailor treatment plans for AIS patients based on their individual characteristics, such as age, curvature severity, and skeletal maturity.	Algorithms for predicting adolescent idiopathic scoliosis treatment	0
Telemedicine and Remote Monitoring: Evaluate the feasibility and effectiveness of telemedicine and remote monitoring technologies in the management of AIS, especially in areas with limited access to specialized care.	Telemedicine and adolescent idiopathic scoliosis treatment	0
Genetic and Molecular Factors: Investigate the genetic and molecular mechanisms underlying AIS. Explore the role of specific genes, genetic markers, or epigenetic factors in the development and progression of scoliosis. This could involve genome-wide association studies (GWAS) or molecular biology techniques.	Genetic and molecular factors of adolescent idiopathic scoliosis	5
Biomarkers for Early Detection: Develop and validate biomarkers that can aid in the early detection and risk assessment of AIS. This could involve the identification of specific proteins, genetic markers, or imaging-based markers that are indicative of scoliosis development.	Biomarkers for early detection of adolescent idiopathic scoliosis	5
Biomechanics and Spinal Growth: Study the biomechanics of spinal growth in adolescents to better understand how abnormal spinal curvature develops. Investigate factors like muscle imbalances, asymmetric loading, and mechanical stressors that may contribute to AIS.	Biomechanics and spinal growth for adolescent idiopathic scoliosis	5
Bracing Efficacy and Optimization: Conduct research to assess the effectiveness of different bracing techniques and protocols in managing AIS. Explore ways to optimize bracing design, duration, and patient compliance to achieve the best outcomes.	Bracing efficacy and optimization for adolescent idiopathic scoliosis	5
Non-Surgical Treatment Modalities: Evaluate the effectiveness of alternative treatments such as physical therapy, scoliosis-specific exercises, chiropractic care, or acupuncture in managing AIS. Compare their outcomes with traditional treatments.	Non-surgical interventions for adolescent idiopathic scoliosis	5
Psychosocial Impact: Investigate the psychosocial impact of AIS on adolescents. Explore the emotional and social challenges faced by individuals with scoliosis and identify strategies for improving their psychological well-being.	Psychosocial impact of idiopathic scoliosis on adolescents	5
Surgical Interventions: If you have access to a medical facility, you can study the outcomes of various surgical interventions for AIS, including different surgical techniques, instrumentation, and postoperative rehabilitation protocols.	Surgical techniques for adolescent idiopathic scoliosis	5
Long-Term Follow-Up Studies: Conduct long-term follow-up studies to assess the progression of AIS into adulthood and the impact of scoliosis on overall health, including spine-related complications and quality of life.	Long-term follow-up studies on idiopathic adolescent scoliosis	5
Patient Education and Compliance: Study strategies to improve patient education and compliance with treatment recommendations, especially in cases involving brace wear or exercise regimens.	How to improve brace compliance for adolescent idiopathic scoliosis	5
Health Economics and Cost-Effectiveness: Analyze the economic impact of AIS treatment, including the cost-effectiveness of various interventions, to inform healthcare policy decisions.	Economic impact of adolescent idiopathic scoliosis	5

**Table 2:** Results from the Gemini query AIS - adolescent idiopathic scoliosis

Gemini responses	PubMed input	Originality score
Genetic Factors: Deeper exploration of specific gene mutations and their interactions in AIS development.	Gene mutations linked to adolescent idiopathic scoliosis	5
Biomechanical Factors: Investigating the role of muscle imbalances, postural habits, and biomechanical stress in AIS progression.	Biomechanical stress in the progression of adolescent idiopathic scoliosis	5
Neuromuscular Factors: Studying the potential involvement of the nervous system in AIS, including vestibular system dysfunction and proprioceptive deficits.	Neurological, vestibular, and proprioceptive deficits in adolescent idiopathic scoliosis	1
Early Detection: Developing more accurate and accessible screening tools for early detection of AIS.	Screening tools for early detection of adolescent idiopathic scoliosis	5
Prognostic Factors: Identifying reliable predictors of AIS progression to refine treatment decisions.	Prognostic factors and predictors of adolescent idiopathic scoliosis progression in treatment decisions	4
Imaging Analysis: Exploring advanced imaging techniques (e.g., MRI, CT, or 3D modeling) to improve curve assessment and treatment planning.	Imaging in the assessment for adolescent idiopathic scoliosis	5
Bracing: Optimizing brace design, fitting, and compliance to improve treatment outcomes.	Optimizing brace design and fitting to improve treatment of adolescent idiopathic scoliosis	2
Exercise and Physical Therapy: Investigating the effectiveness of specific exercise programs in preventing or slowing AIS progression.	Effectiveness of exercise in preventing adolescent idiopathic scoliosis progression	5
Surgical Treatment: Developing less invasive surgical techniques and improving postoperative outcomes.	Minimally invasive surgery for adolescent idiopathic scoliosis	5
Pain Management: Exploring non-pharmacological and pharmacological interventions for AIS-related pain.	Relieving pain from adolescent idiopathic scoliosis	5
Psychosocial Impact: Investigating the psychological and social effects of AIS on adolescents and their families.	Psychosocial effects of adolescent idiopathic scoliosis on patients and their families	3
Quality of Life: Assessing the long-term impact of AIS on quality of life, including physical, emotional, and social well-being.	Long-term impact of adolescent idiopathic scoliosis on quality of life and well-being	4
Adult Outcomes: Studying the long-term consequences of AIS, such as back pain, respiratory function, and functional limitations.	Long-term health consequences of adolescent idiopathic scoliosis	5
Cost-Effectiveness: Evaluating the cost-effectiveness of different treatment options for AIS.	Cost-effectiveness of different treatments for adolescent idiopathic scoliosis	4

**Table 3:** Results from the Copilot query AIS - adolescent idiopathic scoliosis

Copilot responses	PubMed input	Originality score
Investigate the genetic basis of AIS. This could involve identifying specific genes associated with the condition and understanding how genetic variations contribute to the development and progression of scoliosis.	Genetic factors associated with the development and progression of adolescent idiopathic scoliosis	5
Study the biomechanics of the spine in adolescents with AIS. This could include analyzing how different forces and movements affect spinal curvature and identifying potential mechanical factors that contribute to the progression of scoliosis.	Movements that affect spinal curvature contributing to adolescent idiopathic scoliosis	4
Evaluate the effectiveness of various screening methods for early detection of AIS. This could involve comparing traditional methods like the Adam’s forward bend test with newer technologies such as 3D imaging and genetic screening tests.	Traditional and novel screening methods for early detection of adolescent idiopathic scoliosis	5
Research the efficacy of non-surgical treatments such as bracing, physical therapy, and chiropractic care. This could include long-term studies to determine the best practices for managing AIS without surgery.	Long-term conservative treatment for adolescent idiopathic scoliosis	5
Analyze the outcomes of different surgical techniques used to treat AIS. This could involve comparing the long-term results of various procedures, such as spinal fusion and vertebral body tethering, and identifying factors that influence surgical success.	Surgical procedures for the treatment and management of adolescent idiopathic scoliosis	5
Examine the psychosocial effects of AIS on adolescents. This could include studying the impact of scoliosis on self-esteem, body image, and mental health, as well as the effectiveness of support groups and counseling.	Psychosocial effects and counseling for adolescent idiopathic scoliosis	3
Explore the use of new technologies in the diagnosis and treatment of AIS. This could involve researching the application of robotics, artificial intelligence, and virtual reality in surgical planning and rehabilitation.	Use of artificial intelligence and machines in adolescent idiopathic scoliosis	5
Conduct long-term studies to track the natural history of AIS from adolescence into adulthood. This could help identify factors that predict progression and outcomes, and inform guidelines for monitoring and treatment.	Adolescent idiopathic scoliosis into adulthood	3
Investigate the role of environmental factors, such as nutrition, physical activity, and lifestyle, in the development and progression of AIS. This could help identify modifiable risk factors and preventive strategies.	Environmental factors in adolescent idiopathic scoliosis	5
Develop and evaluate educational programs aimed at increasing awareness and understanding of AIS among patients, families, and healthcare providers. This could include creating resources that explain the condition, treatment options, and the importance of early detection.	Strengthening patient education and awareness of adolescent idiopathic scoliosis	0

**Table 4:** Results from the Llama query AIS - adolescent idiopathic scoliosis

Llama responses	PubMed input	Originality score
Genetic predisposition: Investigate specific genetic markers and their association with AIS susceptibility and severity.	Genetic factors associated with the susceptibility and severity of adolescent idiopathic scoliosis	5
Hormonal influences: Examine the role of hormonal fluctuations during puberty in AIS development and progression.	Pubertal hormone fluctuations associated with the development and progression of adolescent idiopathic scoliosis	0
Environmental factors: Study the impact of lifestyle factors, such as physical activity, nutrition, and sleep patterns, on AIS risk and severity.	Environmental factors in adolescent idiopathic scoliosis	5
Early detection methods: Develop and evaluate novel screening tools for early AIS detection, such as AI-powered radiographic analysis or non-invasive biomarkers.	Screening and biomarkers for early detection of adolescent idiopathic scoliosis	5
Predictive modeling: Create predictive models to identify high-risk patients and forecast curve progression.	Models used to identify patients at high risk for developing adolescent idiopathic scoliosis	3
Bracing effectiveness: Conduct randomized controlled trials to evaluate the efficacy of different bracing techniques and materials.	Efficacy of bracing in the treatment and intervention of adolescent idiopathic scoliosis	5
Surgical innovations: Investigate the outcomes and complications of novel surgical techniques, such as robotic-assisted surgery or motion-preserving procedures.	Novel surgical techniques in the outcome and management of adolescent idiopathic scoliosis	3
Non-surgical interventions: Examine the effectiveness of alternative therapies, like physical therapy, chiropractic care, or Schroth method exercises.	Effectiveness of therapy for management and treatment of adolescent idiopathic scoliosis	5
Patient-reported outcomes: Develop and validate AIS-specific patient-reported outcome measures to assess quality of life and treatment satisfaction.	Patient reports of quality of life following treatment of adolescent idiopathic scoliosis	5
Long-term follow-up: Conduct longitudinal studies to evaluate the long-term effects of AIS and its treatment on patients' lives.	Longitudinal studies of chronic effects of adolescent idiopathic scoliosis	5
Spinal biomechanics: Investigate the biomechanical mechanisms underlying AIS, including spinal flexibility, stiffness, and loading patterns.	Physical and Biomechanical mechanisms underlying adolescent idiopathic scoliosis	4
Cellular and molecular biology: Study the cellular and molecular changes occurring in the spine during AIS development and progression.	Cellular and molecular changes in the spine during the development of adolescent idiopathic scoliosis	1

**Table 5:** Tabulation of percent novelty and mean originality score of each AI model

Artificial intelligence model	Number of results generated	Percent novelty	Mean originality dcore
ChatGPT	12	16.67%	4.2 ± 1.9
Gemini	14	0.00%	4.1 ± 1.3
Copilot	10	10.00%	4.0 ± 1.6
Llama	12	8.33%	3.8 ± 1.7

The percent novelty was also calculated for each AI model by calculating the proportion of novel research prompts yielded (indicated by an originality score of 0). ChatGPT had the highest percentage of novel ideas with 16.67% of the prompts being original. Copilot and Llama followed, with 10.00% and 8.33%, respectively, and Gemini yielded no original prompts. 

## Discussion

The results of our study demonstrate that these AI models have the capability of generating novel research ideas in orthopedics. The effectiveness of each model in generating novel research ideas was quantified by the percent novelty, which was highest for ChatGPT (16.67%). Two of the twelve ideas by ChatGPT were completely novel, whereas the other ten were already thoroughly explored; interestingly, there were no prompts with scores between zero and five. Copilot and Llama trailed behind ChatGPT in percent novelty (10.00% and 8.33%, respectively), each yielding one completely novel research idea. None of the fourteen ideas generated by Gemini were completely novel. On average, Llama yielded topics with the most originality, with a mean originality score of 3.8 ± 1.7, while the other platforms all yielded an originality score greater than four (ChatGPT 4.2 ± 1.9, Gemini 4.1 ± 1.3, and Copilot 4.0 ± 1.6). ChatGPT had both the highest percent novelty and the highest mean originality score. 

AI has been applied across a broad range of fields, including banking, manufacturing, and education. In recent years, an increasing number of studies have highlighted the potential of AI platforms such as ChatGPT, Gemini, Copilot, and Llama in academic and clinical medicine. For example, all four models have shown their ability to provide patient education materials and answer medical questions from patients undergoing cardiac catheterization [17]. Additionally, a separate study demonstrated the effectiveness of ChatGPT as a reliable complementary tool for physicians managing obstructive sleep apnea [18]. AI has also shown promise in enhancing discharge summaries, addressing the common issue in traditional discharge methods of missing important details [19-20]. 

Beyond clinical medicine, AI has made significant strides in its role in clinical research. One recent study highlighted how large companies like Google and Apple have leveraged AI to create contract-tracing platforms [21]. Another study found that AI-generated abstracts were nearly indistinguishable from those written by humans, suggesting that AI output detectors could serve as a useful tool for editors in distinguishing between AI and human-authored content [22]. Despite the growing body of research showcasing the utility of AI in medicine and medical research, to date, no study has yet explored its effectiveness in generating novel research ideas in orthopedic surgery. Our study contributes to the existing literature by demonstrating that AI models are capable of generating novel research ideas in orthopedic surgery. 

Despite the impressive capabilities of these AI models and their impressive potential in academic and clinical research, their applicability remains hindered by various shortcomings. One significant issue is their inability to refine their responses to an appropriate target population. For example, in the previously mentioned study on cardiac catheterization, while the information provided by all four AI models was accurate, it was presented at a high school to college reading level, far above the recommended sixth-grade reading level for patient education [17]. This underscores that, although AI models may be useful in assisting with tasks such as generating novel research ideas, they cannot yet be relied on to complete the task independently. Another limitation is the risk of "AI Hallucination", where AI models may present misinformation as fact or even cite false or non-existent sources [11, 23-24]. While this issue is not as prominent in our study, as we are leveraging AI's creative abilities rather than relying on its factual knowledge, it remains a challenge for broader applications. Despite these limitations, our study is the first to evaluate the potential of various AI platforms in generating novel research ideas in the field of orthopedic surgery. 

## Conclusions

Artificial intelligence is a constantly evolving and ever-expanding tool that has wide-reaching applications in both the clinical and academic aspects of medicine. A multitude of studies have demonstrated their effectiveness in complementing physicians in patient care and patient education and even offering their own contributions in research, such as generating literature reviews. Our study is the first to assess the effectiveness of AI in generating novel research ideas in orthopedics and orthopedic surgery. Our findings demonstrate that ChatGPT, Llama, and Copilot are capable of generating novel ideas in orthopedics research. As these models continue to evolve and become even more refined with time, physicians and scientists should consider incorporating them when brainstorming and planning their research studies. 
